# Calcium signalling in weeds under herbicide stress: An outlook

**DOI:** 10.3389/fpls.2023.1135845

**Published:** 2023-03-24

**Authors:** Katerina Hamouzová, Madhab Kumar Sen, Rohit Bharati, Pavlína Košnarová, Md Rafique Ahasan Chawdhery, Amit Roy, Josef Soukup

**Affiliations:** ^1^ Department of Agroecology and Crop Production, Faculty of Agrobiology, Food and Natural Resources, Czech University of Life Sciences Prague, Prague, Czechia; ^2^ Excellent Team for Mitigation (E.T.M.), Faculty of Forestry and Wood Sciences, Czech University of Life Sciences Prague, Prague, Czechia; ^3^ Department of Crop Sciences and Agroforestry, The Faculty of Tropical AgriSciences, Czech University of Life Sciences Prague, Prague, Czechia

**Keywords:** abiotic stress, calcium signalling, food security, herbicide resistance, weeds

## Abstract

The continuous use of herbicides for controlling weeds has led to the evolution of resistance to all major herbicidal modes of action globally. Every year, new cases of herbicide resistance are reported. Resistance is still in progress in many species, which must be stopped before it becomes a worldwide concern. Several herbicides are known to cause stressful conditions that resemble plant abiotic stresses. Variation in intracellular calcium (Ca^2+^) concentration is a primary event in a wide range of biological processes in plants, including adaptation to various biotic and abiotic stresses. Ca^2+^ acts as a secondary messenger, connecting various environmental stimuli to different biological processes, especially during stress rejoindering in plants. Even though many studies involving Ca^2+^ signalling in plants have been published, there have been no studies on the roles of Ca^2+^ signalling in herbicide stress response. Hence, this mini-review will highlight the possible sensing and molecular communication *via* Ca^2+^ signals in weeds under herbicide stress. It will also discuss some critical points regarding integrating the sensing mechanisms of multiple stress conditions and subsequent molecular communication. These signalling responses must be addressed in the future, enabling researchers to discover new herbicidal targets.

## 1 Introduction

Plants cells are the depot for different ions, including calcium. Calcium (Ca^+2^) is an essential bivalent cation with varying plant utilities (Aldon et al., 2018; Yadav et al., 2022). Calcium ions (Ca^2+^) partake in several physiological parameters in plants, including cell division, cytoplasmic streaming, thigmotropism, photomorphogenesis, cell polarization, fruit development and ripening and plant microbe interaction (Gao et al., 2019; Furuichi, 2020; Eichstädt et al., 2021; Duo et al., 2022). Since the discovery of its effects on muscle contraction, the perception of calcium as a regulatory molecule in living organisms has gained wide-range recognition globally. Plant and animal cells contain a group of proteins with Ca^2+^-binding properties (Bose et al., 2011; Jia et al., 2022). These proteins alter their conformation upon stress by external stimulus, increasing the cytoplasmic Ca^2+^ ([Ca^2+^]_cyt_) concentration. Thereafter, the [Ca^2+^]_cyt_ couples the extracellular stimuli to their distinctive intracellular responses and synchronize a wide range of endogenous processes. Besides increasing the concentration of the [Ca^2+^]_cyt_, sometimes these special Ca^2+^-binding proteins directly interact with other targets and allow them to execute their respective response (Batistič and Kudla, 2009). Several shreds of evidence have established the ubiquitous role of Ca^2+^ as an essential cellular second messenger (Marcec et al., 2019). The details on the characteristics and molecular mechanisms of calcium signalling can be found in Bickerton and Pittman, 2012 and Kudla et al., 2018.

The importance of herbicides to modern crop production is immutable. However, due to excessive application of synthetic herbicides, there has been a continuous rise in the number of cases of herbicide-resistant weeds. The evolution of herbicide resistance in weeds is an exceptional example of the adaptability of weed species to abiotic stress (Délye et al., 2013). An increase in cellular Ca^2+^ concentration is a primary signalling event when plants confront abiotic stimuli (Xu et al., 2022). Hence, assuming that calcium signalling might play a vital role in weeds toward herbicidal resistance/tolerance under herbicide stress would not be erroneous. However, to date, there have been no attempts to explore the plausible role of Ca^2+^ in this area (**Supplementary Figure 1**). Hence, in this perspective, we will discuss the plausible roles of Ca^2+^ signalling in conferring herbicide resistance. Because of space limitations, we will cover the main aspects of calcium as a signal transducer involved in herbicide stresses.

## 2 Ca^2+^ signalling in abiotic stresses in plants

Among the vital environmental stresses affecting agriculture, the most important are drought, salt, and temperature stresses (Hirayama and Shinozaki, 2010; Chaudhry and Sidhu, 2022; Zhang H. et al., 2022). To overcome these constraints, plants developed sophisticated mechanisms, including systematic regulation of Ca^2+^ ions within the cells, which might lead to intonations of gene expression (Reddy et al., 2011; Manishankar et al., 2018). Studies suggest Ca^2+^ plays a crucial role in regulating the physiological response to drought conditions by acting as a secondary messenger and transmitting drought signals (Hong-Bo et al., 2008; Pathak et al., 2020). It has also been elucidated that cytosolic-free calcium plays a pivotal role in stomatal movement, and changes in its concentration can regulate the opening and closing of the stomata (Wang et al., 2005; Wang et al., 2016; Thor et al., 2020). The movement of stomata regulates two of the key plant physiological processes, i.e., transpiration and stomatal conductance, which is closely related to water-use efficiency, a physiological trait of immense importance concerning drought stress. Previously, the close relationship between Ca^2+^ concentration and the degree of drought stress in wheat seedlings was examined, and it was observed that the concentration of free Ca^2+^ in the nucleus increases with increasing drought duration, indicating its potential role in maintaining nucleus structure and integrity (Hong-Bo et al., 2008; Song et al., 2008).

Ca^2+^ ions not only promote plant growth but also possess the potency to reverse the detrimental effects of salt stress (Roy et al., 2019; Borjigin et al., 2021; Lian et al., 2022). For instance, salinity causes a severe reduction of hydraulic conductivity in the primary roots of the plants. However, exogenous application of Ca^2+^ mitigated these effects in maize and other plants (Ahmad et al., 2018; Pathak et al., 2020; Lian et al., 2022). Evidence also suggests that the Ca^2+^ signalling network is closely associated with the SOS (salt overly sensitive) signal transduction pathway and regulates the homeostasis of cellular Na^+^ and K^+^ (Brini et al., 2007).

Apart from salt and drought stresses, there has been a significant increase in research correlating Ca^2+^ ions and temperature stress tolerance. Ca^2+^ has been found to play a vital role in plants’ adaptation to cold and heat stress conditions (Hong-Bo et al., 2008; Pathak et al., 2020). For instance, in an attempt to acclimatize in the alpine subnivean conditions, *Chorispora bungeana* accumulates Ca^2+^ among its various tissues and organs (Fu et al., 2006). Ca^2+^-responsive protein kinases such as soybean calmodulin (GmCaM4) and Ca‐dependent protein kinase 6 (CDPK6) in *Arabidopsis* were found to regulate the metabolism of plant cells positively and confer cold tolerance (Pathak et al., 2020). In addition, exogenous treatment of Ca^2+^ during cold stress enhances plant growth, development, morphology, and physiology by modulating the plant’s photosynthetic capacity, ROS metabolism, and nitrogen assimilation (Yuan et al., 2018; Singh et al., 2022). In acclamatory response to heat stress, Ca^2+^ signalling causes increased expression levels of the gene coding for an enzyme called desaturase, an essential enzyme responsible for maintaining membrane fluidity under temperature stress (Huda et al., 2013). Ca-mediated heat shock proteins (HSPs) are also induced during heat stress and provide necessary cellular homeostasis (Pathak et al., 2020). In a previous study on tobacco, the heat stress caused a decrease in various photosynthetic parameters such as net photosynthetic rate, apparent quantum yield and stomatal conductance. However, the exogenous application of CaCl_2_ mitigated these effects (Tan et al., 2011). In the same study, activities of ascorbate peroxidase, superoxide dismutase, catalase, and peroxidase also decreased under heat stress, but with CaCl_2_ pretreatment, the levels of these enzymes showed significant alterations (Tan et al., 2011). Hydrogen peroxide (H_2_O_2_) tends to accumulate in plants as a response to stress like high temperatures and has been extensively used as a physiological indicator to assess the intensity of stress felt by plants (Asaeda et al., 2022). Interestingly, the levels of H_2_O_2_ have also been found to decrease upon exogenous calcium chloride treatment indicating enfeebling effects of Ca^2+^ during stresses (Tan et al., 2011; Pathak et al., 2020). **Table 1** lists some critical Ca^2+^ signalling in abiotic stress-specific research works identified in major plant species in the last few years.

**Table 1:** Some important Ca^2+^ signalling in abiotic stress-specific research works identified in major plant species.

Serial number	Species	Experimental condition	Comments	Reference
Temperature stress response				
1	*Davidia involucrata*	Heat stress response	The authors found that the overexpression of DiATG3 (an autophagy-related gene) in arabidopsis activated the calcium signaling and mitogen-activated protein kinase (MAPK) signaling pathways, which in turn conferred thermotolerance and enhanced autophagy under heat stress.	Liu et al., 2023
2	*Oryza sativa*	Heat and cold tolerance	In this study, the authors found that under temperature-stress tolerance in rice, the loss-of-function of two cyclic nucleotide-gated ion channel proteins (OsCNGC14 and OsCNGC16) displayed reduced survival rates and fitness.	Cui et al., 2020
3	*Nicotiana benthamiana*	Cold tolerance	In this study, the authors overexpressed a *CDPK* gene (*MdCPK1a*; isolated from apple) in *Nicotiana benthamiana* and found that the overexpressed tobacco plants performed better than the wild type under cold stress. They also investigated the underlying molecular mechanisms and found that this better adaptability is due to the accumulation of scavenging ROS and adjustments in the expression pattern of stress-related genes.	Dong et al., 2020
4	*Arabidopsis thaliana*		Under cold stress, the OST1 kinase activates and promotes ANNEXIN1-dependent calcium signalling. When AtANN1 is phosphorylated, its Ca^2+^ transport activity increases, resulting in enhanced Ca2+ signalling. Finally, OST1-AtANN1 regulates the expression of cold-responsive genes (*CBF* and *COR*).	Liu et al., 2021
5			In this experiment, the authors identified that MCA1 and MCA2 (calcium-permeable mechanosensitive channels) contributes to the cold tolerance in Arabidopsis. The authors found that MCA1 and MCA2 mutants exhibit lower cold tolerance than the wild type.	Mori et al., 2018
Salt stress response				
6	*Arabidopsis thaliana*	Salt stress response	In this study, the authors provided evidence that CBL10 (a calcium sensor protein) acts as an interconnecting regulator and synchronizes the plant’s response under saline and alkaline stresses.	Xie et al., 2022
7			The authors had shown that, in arabidopsis, under salt stress, AtANN4 (a calcium-permeable transporter), along with its interacting proteins (SCaBP8 and SOS2), create a calcium signal. This calcium signal signature activates the SOS pathway, modulating ion homeostasis under salt stress.	Ma et al., 2019
8			In this study, the authors have shown that 14-3-3 proteins (a Ca^2+^-dependent molecular switch) regulate the plant’s response to salt stress. They proposed that 14-3-3 proteins regulate the SOS2 and PKS5 proteins. These proteins, in turn, activate the PM Na^+^/H^+^ antiporter and the PM H^+^-ATPase (both of which are important in salt stress regulation).	Yang et al., 2019
9			The authors found that differential expression of calcineurin B-like 10 (CBL10) contributes significantly and increases resistance to salt stress conditions.	Kang and Nam, 2016
10			The authors discovered that a Na^+^/Ca^2+^ exchanger-like protein, encoded by Arabidopsis NCX-like (AtNCL) is involved in salt stress in arabidopsis.	Wang et al., 2012
11	*Actinidia valvata*		In this study, the authors tried to characterize the *CIPK* gene family members under salt stress. Based on the transcriptome data, the authors identified 42 *CIPK* genes in *A. valvata*. Furthermore, the differential expression analysis pattern showed that overexpression of *AvCIPK11* is associated with heightened salt tolerance.	Gu et al., 2023
12	*Zea mays*		In a phosphoproteomic analysis with two contrasting maize inbred lines, the authors found the possible involvement of the calcium/proton exchanger CAX1-like protein in initiating the salt signal transduction pathway.	Zhao et al., 2019
Drought stress response				
13	*Arabidopsis thaliana* *Arabidopsis thaliana*	Drought stress response	The authors provided evidence that antagonistic activities of two Ca^2+^ sensor proteins, CML37 and CML42, alter phytohormone signalling and confer adaptation under drought and herbivory stress. The authors also showed that the exact mechanism provides defence against *Alternaria brassicicola* (a necrotrophic pathogen).	Heyer et al., 2022
14			The authors report that CPK8 (calcium-dependent protein kinase 8) plays important regulatory roles in abscisic acid (ABA)- and Ca^2+^-mediated plant responses to drought stress. They found that CPK8 mutants are more sensitive to drought stress than the wild-type plants; however, transgenic plants with overexpressed CPK8 exhibited improved drought tolerance.	Zou et al., 2015
15	*Zea mays*		The authors showed that ZmCIPK8 (a member of plant CBL-interacting protein kinases) regulates the stress-related genes during water deficiency.	Tai et al., 2016
16			In their study, the authors identified that Ca^2+^-dependent protein kinases ZmCPK35 and ZmCPK37 were expressed in maize guard cells and played essential roles in stomatal closure during drought stress.	Li et al., 2022
17	*Nicotiana tabacum*		In this study, the authors screened combinatorially transformed tobacco plant lines and identified that a combination of calcium-dependent protein kinase genes might enhance the plant’s growth under limiting water conditions. To test their hypothesis, they introduced the targeted gene combination *via* genetic engineering and discovered that it increased plant survival rates under drought stress.	Schulz et al., 2021
Other stresses				
18	*Arabidopsis thaliana*	K^+^ deficiency	The authors discovered that a Ca^2+^-dependent signalling network plays important regulatory roles during K^+^ starvation. A more in-depth investigation revealed that this Ca^2+^-dependent signalling network influences vacuolar K^+^ remobilization rather than regulating K^+^ uptake.	Tang et al., 2020
19			The limited availability of free potassium ions in soil imposes persistent stress on plants. The researchers discussed how a Ca^2+^-dependent signalling network plays critical roles in plants’ response to K^+^ deficiency	Wang Y. et al., 2021
20			The authors identified that, upon being stressed by K^+^ deficiency, two consecutive and different Ca^2+^ signals are generated in the roots of arabidopsis. Further investigation of the detailed mechanism using patch-clamp and TEVC analyses discovered that AKT1 (a K^+^ channel) gets activated *via* the CBL1–CIPK23 Ca^2+^ sensor-kinase complex.	Behera et al., 2017
21		Mechanical stress	The authors demonstrated the possible involvement of Ca^2+^ signalling in mechanically stimulated arabidopsis *via* receptor-like kinase FERONIA (FER).	Shih et al., 2014
22		Nitrate stress	The authors used aequorin reporter plants to demonstrate that treating nitrates might momentarily increase cytoplasmic Ca^2+^ concentration in nitrate-starved arabidopsis roots.	Riveras et al., 2015
23		Osmosensing	While studying arabidopsis OSCA1 mutants, the authors found that OSCA1 (a hyperosmolality-gated calcium-permeable channel) is important in osmosensing in plants when induced by a stimulus.	Yuan et al., 2014
24	*Oryza sativa*	Arsenic stress	The authors found that when arsenic-exposed rice seedlings were compensated by calcium, they demonstrated a significant reduction in ROS production and a significant increase in antioxidant enzyme activities compared with seedlings exposed to arsenic only. The authors concluded that the calcium might be associated with arsenic stress mitigation in rice.	Rahman et al., 2015
25	*Zea mays*	Oxidation stress	The authors identified that calcium (Ca^2+^)/calmodulin (CaM) mediated signalling is involved in ABA-induced antioxidant defense in maize. The researchers further conducted transient expression studies and RNAi-based experiments with maize Ca^2+^/CaM-dependent protein kinase (ZmCCaMK) and confirmed that ZmCCaMK is required for ABA-induced antioxidant defense.	Ma et al., 2012

## 3 Ca^2+^ signalling might lead to transcriptional reprogramming during herbicide stress

The most basic molecular mechanisms of herbicide resistance include target-site resistance (TSR) and non-target-site resistance (NTSR). Among the TSR, genetic mutations and gene overexpression are the most prevalent mechanisms. NTSR mechanisms include enhanced metabolism, reduced absorption and translocation etc. (Gaines et al., 2020; Torra and Alcántara-de la Cruz, 2022). NTSR mechanisms are more complex than TSR mechanisms and involve transcriptional reprogramming of stress-related genes such as *cytochrome P450s* (*CYP450s*) and *glutathione-S-transferases* (*GSTs*). Hence, the development of herbicide resistance in grass weeds can be considered almost similar to other abiotic stresses such as temperature and drought. Weedy plants have developed several exciting mechanisms to adapt against herbicide stress. Although numerous mechanisms have been discovered, several such mechanisms, especially different signalling events, are yet to be discovered.

Ca^2+^ signalling events amend the expression patterns of several essential genes (mainly NTSR genes) in response to several biotic and abiotic stresses (Moscatiello et al., 2018; Yadav et al., 2022). Depending upon the activation mechanisms, Ca^2+^ influx transporter channels might translate a wide range of signals into diverse Ca^2+^ signatures. To date, five different families of Ca^2+^ channels have been recognized in higher plants. These include two-pore channels (TPCs), glutamate receptor-like channels (GLRs), Cyclic nucleotide-gated channels (CNGCs), mechanosensitive-like channels (MSLs) and the reduced hyperosmolality-induced [Ca^2+^]_cyt_ increase (OSCAs) channels. Within the plant cells, these channels are maintained precisely in an extremely synergistic manner (Ghosh et al., 2022). Upon being stressed by herbicides, it could be assumed that these Ca^2+^ influx transporters might get triggered, and hence there might be a sudden influx of Ca^2+^, thereby creating herbicide stress-specific Ca^2+^ signatures. Thereafter, the diverse and extensive set of Ca^2+^ sensors (known as Ca^2+^ binding proteins) decodes and relays the signals for further processing (usually phosphorylation responses). These Ca^2+^ binding proteins can be classified into sensor relay proteins and sensor responder proteins. The sensor responder proteins include calcium-dependent protein kinases (CDPKs), which mainly bind to these Ca^2+^ ions and induce conformational changes (Hashimoto and Kudla, 2011). After that, the herbicide-stress-specific CDPKs decode and translate the message of elevated calcium concentration into enhanced protein kinase activity and subsequent downstream signalling events. Contrary to the responder proteins, the relay proteins lack effector domains and are not direct target proteins. The relay proteins include calmodulins (CaMs), and their primary function is to bind to these Ca^2+^ ions and induce conformational changes in the responder proteins (Hashimoto and Kudla, 2011).

Although the lack of higher-resolution genomic data and tools in weeds have limited our detailed knowledge of the CDPKs in these species, these proteins are well-characterized for the model plants such as *A. thaliana* (Shi et al., 2018). Additionally, molecular cloning and functional analysis studies have confirmed the essential roles of CPKs in abiotic stress tolerance in a wide range of plants (Atif et al., 2019). CDPKs contain a serine/threonine protein kinase domain, an autoinhibitory domain, and a CaM-like domain. The autoinhibitory domain plays a vital role in maintaining the activated state of the kinase domain. Once the cellular Ca^2+^ levels get elevated, the binding of Ca^2+^ to the CaM-like domain leads to a conformational change of the autoinhibitory domain and activates the kinase. In addition to the conformational changes of the CaM-like domain, auto-phosphorylation of these proteins further helps in the kinase activation process. Along with the CDPKs, plants contain two other sensor responder proteins: Ca^2+^/calmodulin-dependent protein kinases (CCaMKs) and CBL-interacting protein kinases (CIPKs). These kinases and their specific Ca^2+^ binding proteins form a complex cellular network in various cellular processes (Batistič and Kudla, 2009; Weinl and Kudla, 2009). Sometimes these complex cellular networks are stress-specific, and to the best of our knowledge, there are no reports or efforts to discover herbicide-stress-specific cellular signalling systems.

**Figure 1:**
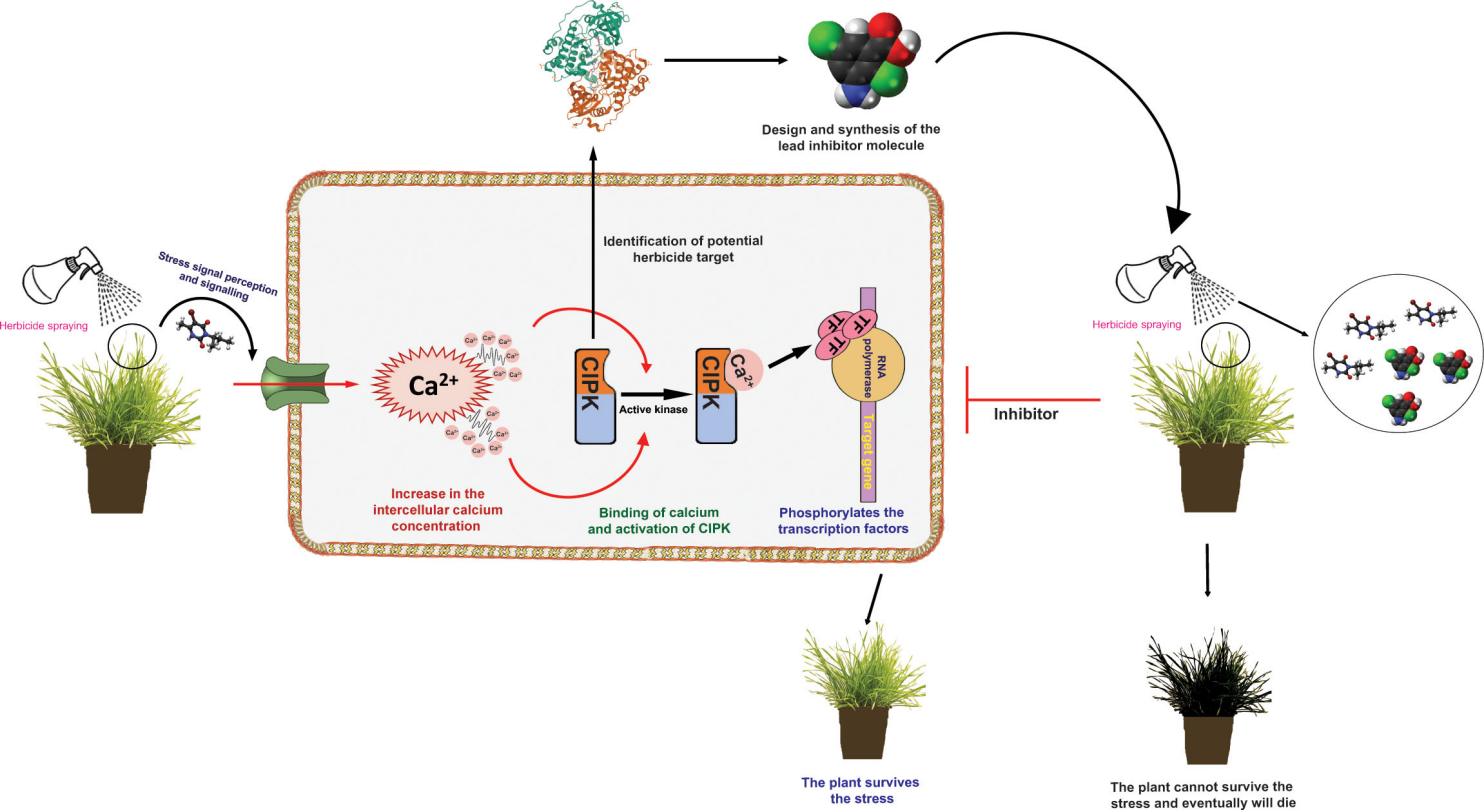
Schematic diagram showing the possible role of calcium (Ca^2+^) signalling in weeds in herbicide resistance. The resistant plant (R) survives the herbicide stress by target site and non-target site mechanisms. The hypothesis is that upon perceiving and transducing the herbicide stress signals, there might be an abnormal elevation of the Ca^2+^ ions within the cytoplasm of the cell. This initiates the calcium signalling pathways. The Ca^2+^ ions are thought to bind to the sensor responder proteins and activate their kinase activities. Instead, these kinases activate the transcription factors by phosphorylating them, thus leading to the overexpression of the stress-responsive metabolism-related genes. In turn, these genes enhance the herbicide metabolism process, conferring resistance in R plants. Following the identification and validation of the potential herbicidal targets (e.g., CIPK isoforms), computer-aided drug design can be used to identify and screen the new inhibitors, which might contribute to the novel herbicidal lead compound discovery.

The final step in calcium signalling under herbicide stress will be converting these complex signals into their respective transcriptional responses. The activation of the Ca^2+^ sensor kinases further induces phosphorylation events, thus leading to the transcriptional reprogramming of essential genes (Khare et al., 2020). Usually, these transcriptional reprogramming events are mediated by activating stress-specific transcription factors (Himanen and Sistonen, 2019). Ca^2+^/CaM-mediated transcription factors relay Ca^2+^ transients generated by herbicide stress to transcriptional reprogramming of *CYP450*s and *GST*s. RNA-seq transcriptome profiling studies have provided shreds of evidence that upregulation of CYP450 isoforms is involved in the metabolism of different herbicides in several weed species, including *Echinochloa* sp. (Fang et al., 2019; Pan et al., 2022), *Alopecurus* sp. (Zhao et al., 2017; Franco-Ortega et al., 2021). However, in most of the cases, the mechanisms of the CYP450 upregulation were not investigated. Transcription factors are potentially involved in regulating the expression of the NTSR genes, such as *CYP450s*, *GSTs* and *ABC transporters*. A recent study by Zhang Y. et al. (2022) on *Echinochloa* spp. has shown that upregulation of the *bZIP88* transcription factor confers resistance to three different herbicides (Zhang Y. et al., 2022). However, the molecular mechanisms behind the upregulation were not further investigated. bZIP transcription factors are known to regulate abiotic stress responses in many plants and are expected to play a regulatory role in NTSR (Century et al., 2008; Zhang H. et al., 2022). Investigations of the calcium signalling events during the herbicide treatment might provide new understandings to such unexplained questions. A schematic diagram showing the putative role of calcium signalling in weeds in herbicide resistance can be found in **Figure 1**.

Besides playing a role in conferring resistance against the herbicides, Ca^2+^ signalling might also play an essential role during stressful “herbicide-exposure memories” in weeds. Higher plants, including weeds, have developed different mechanisms (established over a long period of evolution) to respond and adapt to recurring stresses (Bhar et al., 2022). Among the two distinct mechanisms of herbicide resistance, the NTSR mechanisms play critical roles in integrating and coordinating whole plant herbicide stress responses. There is considerable evidence that [Ca^2+^]_cyt_ signatures get modified by previous experience with an environmental challenge (Bruce et al., 2007). There is also evidence that prior exposure to contrasting stress can alter the Ca^2+^ signatures provoked by a particular stress (Tuteja and Mahajan, 2007). These indicate a cross-talk between the signalling cascades. Frequent exposure to herbicides might lead to an attenuated response of Ca^2+^ signalling pathways, and the cells might retain the previous information. Since maintaining a cellular [Ca^2+^] homeostasis is vital for any plant, this Ca^2+^ memory is significant for herbicide-resistant weeds. These weeds can thus quickly retort better to the herbicide stress without upsetting the delicate and sensitive balance of Ca^2+^.

## 4 Ca^2+^signal-regulated alternative splicing in weeds

Alternative splicing (AS), in general, generates multiple mRNA isoforms from the same pre-mRNA and is alleged to contribute to increasing transcriptomic and proteomic diversity (Chaudhary et al., 2019; Mandadi et al., 2022; Tognacca et al., 2022). The putative roles of AS in a wide range of physiological processes (such as plant metabolism, plant immunity, plant disease resistance etc.) have been well explored in many plants. However, despite the evidence of multiple copies of important herbicide target genes (such as *ALS, ACCase* and *EPSPS*), the area of alternative splicing has remained unexplored in weedy plants (Xu et al., 2014; Iwakami et al., 2017; Yakimowski et al., 2021). Although no experiments have shown AS in weeds, NGS and omics approaches in plants have revealed that most of the plant multi-exon genes undergo AS (Chaudhary et al., 2019). We hypothesize that Ca^2+^-signalling might influence the alternative splicing of these herbicide target genes by controlling the relevant splicing factors. Earlier, we discussed that elevation of the intracellular Ca^2+^ level activates a variety of signalling kinases; after that, the Ca^2+^-binding sensor proteins trigger phosphorylation responses during abiotic stresses (Xu et al., 2022). So, it is possible that when exposed to herbicides, Ca^2+^-binding sensor proteins might stimulate the splicing factors *via* phosphorylation. Furthermore, Ca^2+^-signalling regulates the subcellular redistribution of splicing regulatory proteins (Razanau and Xie, 2013). For example, in rats, thapsigargin treatment triggered an increase in the intracellular calcium concentration, leading to hyperphosphorylation and further accretion of a splicing factor in the cytoplasm (Daoud et al., 2002). Moreover, polarization and depolarization might also significantly impact the chromatin accessibility of the variable exon by regulating the histone modifications (Razanau and Xie, 2013). As a result, Ca^2+^-signalling is thought to play an important role in mRNA isoform expression by controlling chromatin status. In summary, comprehensive experiments are needed to elucidate possible regulatory roles of Ca^2+^-signalling in alternative splicing in herbicide-resistant weeds.

## 5 Monitoring the Ca^2+^ signal patterns associated with herbicide stress response

Even though many indicators are available for plants, none of those have been used in weedy plants to understand the role of Ca^2+^ signalling in alleviating herbicide stress. Since changes in the intracellular and cytosolic Ca^2+^ concentration are stimulus-specific, Jaffe posited three critical criteria to ensure that a particular Ca^2+^ signal pattern is associated with a particular stress response. These criteria include: (a) inhibition of the levels of Ca^2+^ must also impede the physiological response; (b) an artificial increase in the Ca^2+^ levels must provoke the physiological response, even in the absence of the stimulus; and (c) an increase in the cytosolic Ca^2+^ concentration must either precede or accompany the response (Jaffe, 1980). The first two criteria can be accomplished by various chelators and ionophores, whereas the last criterion requires techniques that can measure intracellular resting and stimulated Ca^2+^ levels in weeds. Hence, monitoring the Ca^2+^ signal patterns associated with the herbicide stress response is crucial.

In the past years, Ca^2+^-selective microelectrodes were used to evaluate the modifications of the intracellular Ca^2+^ concentration. However, owing to their convenience, in recent years, these microelectrodes have been replaced by luminous Ca^2+^ indicators, such as Fura Red, Fluo-4, quin-2 and indo-1 (Bush and Jones, 1990; Kanchiswamy et al., 2014). Confocal laser scanning microscopy (CLSM) with these fluorescent Ca^2+^ indicators might be an excellent option for measuring Ca^2+^ in living plant cells. Besides CLSM, calcium imaging can also be done based on Förster Resonance Energy Transfer (FRET) and Selective Plane Illumination Microscopy (SPIM). Even though FRET has been used in *Arabidopsis*, to our knowledge, these techniques have not been used to understand calcium signalling in any weedy plants. In addition to these Ca^2+^ dyes, protein-based Ca^2+^ indicators (such as aequorin–based and GFP–based indicators) are also used to measure Ca^2+^ in living plant cells (Mithöfer and Mazars, 2002; Granatiero et al., 2014; Kanchiswamy et al., 2014). Aequorin is a calcium-activated photoprotein composed of apoaequorin (as apoprotein) and coelenterazine (a luciferin molecule, as a prosthetic group). When Ca^2+^ occupies the Ca^2+^-binding sites of the aequorin, the coelenterazine gets converted into coelenteramide and is released together with carbon dioxide. Thereafter, on returning to its ground state, blue light (λ = 469 nm) is emitted, which can be detected with a luminometer. Bioluminescent probes paired with Ca^2+^-sensitive aequorin can be used in the weeds for real-time measurement of the Ca^2+^ signal patterns linked with herbicide stress. In addition to the aequorin-based Ca^2+^ sensors, since their discovery in 1997, the GFP-based Ca^2+^ indicators have also gained the interest of plant biotechnologists (Kanchiswamy et al., 2014).

## 6 Current challenges in Ca^2+^ signalling in the weeds and the way forward

To understand the mechanisms of Ca^2+^-mediated molecular signalling processes, understanding the expression patterns and correlations with their biochemical activities is vital. Additionally, conducting these analyses in a spatiotemporal fashion will be better. Even though these Ca^2+^-induced changes are well documented in some model plants (Cui et al., 2020; Schulz et al., 2021; Wang J. et al., 2021; Gao et al., 2022; Li et al., 2022), reckonable data at high spatiotemporal resolution are still inadequate. In weeds, to date, there are no attempts to study both Ca^2+^-induced changes and high spatio-temporal resolute data. Elucidation of the molecular signalling mechanisms in model species is mainly based on high-resolution genomic analyses under controlled experimental conditions. Quite the reverse, the unattainability of high-resolution genomic data hampers the identification of the connection between various Ca^2+^ signatures with the phenotypic effects in non-model organisms such as weeds. Additionally, no practical tools for *in-vivo* Ca^2+^ quantification are available for the weed system. However, attempts must be made using existing tools available for Ca^2+^ quantification within the plant system. Additionally, *In-silico* analysis of the gene families involved in the Ca^2+^ signalling will help detect Ca^2+^ transporters in weeds (Goel et al., 2011; Mohanta et al., 2015; Yadav et al., 2022).

## 7 Conclusions and future perspectives

Despite extensive research on the mechanisms of herbicides in recent years, the mechanisms of herbicides and the herbicide stress signalling network have not been investigated and discovered. Much progress has been made in understanding several Ca^2+^-mediated signal networks in crops and plants under various environmental stresses such as cold, heat and drought (Kudla et al., 2018). Similar to plant abiotic stresses, herbicides are known to cause oxidative stress (Caverzan et al., 2019). Hence, the resistance against these chemicals involves the increased expression of several stress-responsive genes, such as CYP450s and GSTs (Délye et al., 2013). Several signal pathways might be involved during herbicide stress signalling (Alberto et al., 2016). In this perspective, we attempted to describe a possible hypothesis on how a Ca^2+^-mediated signal network might confer herbicide resistance. However, due to the lack of any solid experimental attempts evidence, we had to confine ourselves to the theoretical concept as discussed in Section 3 and **Figure 1**. Currently, research efforts are increasing to obtain reference weed genomes. In addition, bioinformatics-based modelling approaches, RNA-seq transcriptome and gene editing experiments (using RNAi and/or CRISPR/Cas systems) are needed to elucidate the exact roles of Ca^2+^ signalling in weeds under herbicide stress. Implementing the multi-omics data-based approaches (generated from genomics and transcriptomics) will be very useful in identifying the candidate Ca^2+^ transducing proteins, channels, exchangers and pumps involved in weed-herbicide interactions and other abiotic stress responses in weeds. Weeds have developed resistance against all the major herbicidal modes of action. Hence, further genetic engineering of these stress-specific Ca^2+^-signalling regulators might enable the herbicide industries to discover novel targets and use them for herbicide discovery. Research on weed molecular communication during herbicide stress (particularly Ca^2+^ signalling) is still in its infancy. This area of research should be an exciting area for future research in terms of novel and applied science.

## Data availability statement

The original contributions presented in the study are included in the article/**Supplementary Material**. Further inquiries can be directed to the corresponding author.

## Author contributions

MS and AR conceived the idea. MS, KH, RB and PK drafted the manuscript. MS and RAC designed and created the figure. AR and JS edited the manuscript MKS revised the manuscript. All authors contributed to the article and approved the submitted version.

## Supplementary material

Supplementary Figure 1 Number of calcium signalling-related publications (2010–2022) retrieved from Web of Science (https://www.webofscience.com/wos/woscc/basic-search). The searches were done by using the keywords “calcium signalling”, “calcium signalling in animals”, “calcium signalling in plants” and “calcium signalling in weedy plants” (done in 10^th^ February, 2023).
