# Supplementary figures and images for: Calcium signalling in weeds under herbicide stress: An outlook
Source: Front Plant Sci. 2023 Mar 24;14:1135845. doi: 10.3389/fpls.2023.1135845 (PMC10080077; doi:10.3389/fpls.2023.1135845)

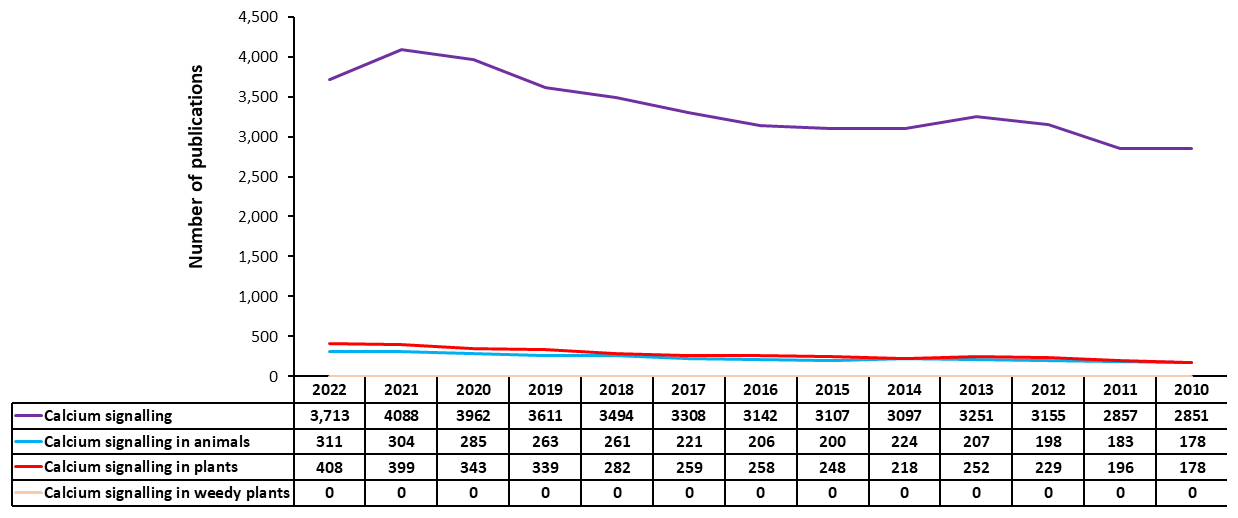

Supplement: Supplementary Figure 1 — Number of calcium signalling-related publications (2010–2022) retrieved from Web of Science (https://www.webofscience.com/wos/woscc/basic-search). The searches were done by using the keywords “calcium signalling”, “calcium signalling in animals”, “calcium signalling in plants” and “calcium signalling in weedy plants” (done in 10th February, 2023). [file Image_1.tif]
